# Combining patch-clamping and fluorescence microscopy for quantitative reconstitution of cellular membrane processes with Giant Suspended Bilayers

**DOI:** 10.1038/s41598-019-43561-4

**Published:** 2019-05-10

**Authors:** Ariana Velasco-Olmo, Julene Ormaetxea Gisasola, Juan Manuel Martinez Galvez, Javier Vera Lillo, Anna V. Shnyrova

**Affiliations:** Biofisika Institute (UPV/EHU, CSIC) and Department of Biochemistry and Molecular Biology, University of the Basque Country, Bilbao, Spain

**Keywords:** Biophysical methods, Membrane structure and assembly

## Abstract

*In vitro* reconstitution and microscopic visualization of membrane processes is an indispensable source of information about a cellular function. Here we describe a conceptionally novel free-standing membrane template that facilitates such quantitative reconstitution of membrane remodelling at different scales. The Giant Suspended Bilayers (GSBs) spontaneously swell from lipid lamella reservoir deposited on microspheres. GSBs attached to the reservoir can be prepared from virtually any lipid composition following a fast procedure. Giant unilamellar vesicles can be further obtained by GSB detachment from the microspheres. The reservoir stabilizes GSB during deformations, mechanical micromanipulations, and fluorescence microscopy observations, while GSB-reservoir boundary enables the exchange of small solutes with GSB interior. These unique properties allow studying macro- and nano-scale membrane deformations, adding membrane-active compounds to both sides of GSB membrane and applying patch-clamp based approaches, thus making GSB a versatile tool for reconstitution and quantification of cellular membrane trafficking events.

## Introduction

Intracellular membrane trafficking exploits the whole range of membrane curvatures. When a transport vesicle buds from a flat parent membrane the membrane curvature gradually increases as the vesicle shapes up into a submicron sphere or cylinder. At the neck of the vesicle extreme membrane curvatures emerge, approaching values similar to the thickness of the lipid bilayer^1^. The curvature stresses ultimately resolve through a local instability leading to membrane fission^1,2^. Different dedicated protein machineries control the vesicle budding, maturation, and fission^3^. Our mechanistic understanding of these machineries is heavily based upon *in vitro* reconstitution of membrane remodelling with purified and/or synthetic molecular components^4^.

Importantly, in the cellular context, the protein machineries operating at different membrane curvatures all work in close coordination to enable seamless and controlled membrane transformation. Yet, only a few *in vitro* membrane templates enable exploring the whole range of membrane curvatures implicated in membrane remodelling *in vitro*. The most widespread system is the giant unilamellar vesicles (GUVs). The micrometric size of these spherical membrane structures mimics perfectly the initial flat curvature of the plasma membrane^5^. Furthermore, GUVs are extremely soft systems easily deformable by distinct protein players, thus allowing dissecting complex membrane deformations into distinct stages and assessing the effects of individual proteins, protein complexes, and lipid species^5–8^. Crucially, the membrane transformations can be observed directly by means of fluorescence microscopy, making GUVs an indispensable template in the analyses of protein-lipid interactions in cellular membrane trafficking^9–14^. Nevertheless, free-floating GUVs are mostly used for ensemble measurements and characterization of collective phenomena (e.g., GUV shape transformation, phase separation and component segregation^15–17^). To visualize and quantify individual membrane remodelling events localized to the nanoscale, GUVs are to be immobilized and controlled.

Numerous methods for GUV immobilization have been described in the literature, such as micro-aspiration^18^ or electrical, optical or microfluidic trapping^19–21^ enabling single-molecule analyses^22^. Yet, robust non-invasive immobilization of GUV remains a methodological challenge. Even when immobilized, GUVs remain closed membrane systems virtually lacking membrane reservoir, which is stored only in the entropic undulations of the membrane^18^. Upon addition of a remodelling protein to the GUV membrane, this reservoir is rapidly exhausted building up the lateral membrane tension, which interferes with the protein activities and increases the risk of membrane rupture^11,23^. The GUV closeness also leads to different non-local effects (e.g., non-local elastic response^15^), making the whole GUV membrane responsive to local membrane perturbation^23^. Both, effective membrane tension and non-local effects in the GUV system can be regulated by supplementing GUVs with an ad hoc membrane reservoir. For example, the integral robustness of the GUV template can be increased by creating an extra membrane reservoir, as in the case of inward-facing nanotubules induced by a compositional asymmetry of the GUV membrane^23^. Similar internal membrane nano-reservoirs have been implicated in controlling membrane tension and molecular composition in cellular membrane remodelling^24^.

At the microscale, the effect of the reservoir can be modelled by micro-aspiration, where part of the GUV membrane sucked into a micropipette becomes a quasi-reservoir, with the aspiration pressure controlling the lateral membrane tension^18^. Micro-aspiration also immobilizes a GUV, facilitating quantitative microscopy observation of its membrane^11,18,23^. However, only one GUV at a time is assessed, making the experimental analysis tedious. Hence, during the last decades, considerable effort was directed into elaborating alternative methods to prepare micron-scale immobilized membrane templates with controlled membrane reservoir. Many of these new methodologies employ microfluidics and soft-lithography, as well as advanced surface chemistry treatments, the approaches still not readily available in a biochemical lab. Alternatively, substantial progress has been achieved by modifying the protocols of preparation and manipulations of “classical” GUVs. For example, Orwar and co-authors proposed to produce GUVs through rehydration of lipid films deposited directly on a coverslip^25^. This way, upon rehydration, the emerging GUVs remain firmly attached to the multilamellar blob on the coverslip, greatly simplifying micromanipulation and optical monitoring of the membranes. Although the authors did not specifically address this issue, the attachment of the GUVs to the extensive membrane reservoir of the blob should greatly affect the stability of the GUVs, making them more robust than the usually free-standing vesicles^23^. However, while such spontaneous hydration of lamellas has many advantages for GUV preparation, this method is seemingly limited to certain lipid compositions and low salt solutions^26^.

Pucadyil and Schmid developed another approach to specifically target the membrane trafficking events^27^. The Supported Bilayers with Excess Membrane Reservoir (SUPER template) consists of membranes directly supported on the curved surface of a micron-sized silica bead^27^. SUPERs form through osmotic disruption followed by fusion of large unilamellar vesicles on the bead surface. For SUPER preparation, biomimetic lipid compositions containing significant amounts of charged lipid species relevant to endocytic events can be used^27,28^. The limited size of the silica bead reservoir makes outward deformations of the SUPER template readily detectable by optical microscopy. Hence, the SUPER templates have been applied to the real-time analysis of membrane remodelling by specialized proteins, e.g. dynamins^28^. Importantly, as with the attached GUVs developed by Orwar’s group, the immobilization of the SUPER template on the bead greatly facilitates mechanical manipulations of the membrane^28,29^. Unlike the attached GUVs though, the SUPERs can be transferred from one solution to another. This property gives an important extra degree of freedom to the system allowing for biochemical characterization of the membrane remodelling processes^27^. Still, one critical limitation in the SUPER template is a virtual lack of luminal space that substantially restricts the reconstitution and visualization of membrane remodelling, e.g., by fluid fluorescence markers.

Controlled immobilization of GUV or SUPER templates allowed transforming these macroscopic membranes into highly curved nanotemplates, the lipid nanotubes (NTs)^30^. NTs are hollow membrane cylinders (10 to 100 s of nm in diameter), which form when an external force pulls on a reservoir membrane. Since decades ago, GUV-linked NTs have been used to study the mechanics of the lipid bilayer, i.e., the stretching and bending elasticity^30–32^. Importantly, along with the lateral tension, the bending rigidity of the membrane is the major factor in controlling membrane remodelling^30,32^. Besides its important role in membrane elasticity studies, the GUV-NT system has been used to explore the effects of curvature on membrane processes and organization^30,33–35^, as well as to create intricate membrane networks and conduits^35,36^.

The NT is effectively a unidimensional system with its radius being fully defined by its effective bending rigidity and lateral tension^32,37^. Generally, measurements of the NT radius relies on off-line calibration, as this membrane structure is below microscopic resolution^30^. Alternatively, the radius of the NT lumen can be monitored in real time by conductance measurements^37,38^. Although this patch-clamp based approach resolves the radius changes with nanometer precision, so far it has been used only with NTs pulled from horizontal planar lipid bilayers^37^, the configuration precluding simultaneous application of fluorescence microscopy. Recently, a combination of patch-clamp and fluorescence microscopy monitoring was achieved with GUV stabilized by a dynamic passivation mechanism^39,40^. However, GUV fragility and lack of electrical connectivity between GUV interior and exterior preclude the detection of nanoscale remodelling of GUV and/or NT membranes.

Here we solve these challenges by combining some of the recent developments in GUV production with the SUPER template technique. We found that gentle hydration of lamellas deposited on silica beads, similar to that used in SUPER templates, leads to the formation of GUV-like structures attached to the bead. We call these structures Giant Supported Bilayers (GSBs). The GSB template possesses several novel features, such as overall robustness given by a big membrane reservoir, virtually no limitations in membrane compositions and real-time access granted to both membrane leaflets. GSBs can also be used as a reservoir for NTs, allowing for their simultaneous electrical and microscopy-based monitoring. Finally, GSBs can be transformed into classical GUVs by detaching them from the beads, thus allowing GUV production in a fast (tens of minutes) and robust way from virtually any lipid composition.

## Results and Discussion

### Surface curvature promotes swelling of membrane lamellas

**Figure 1:**
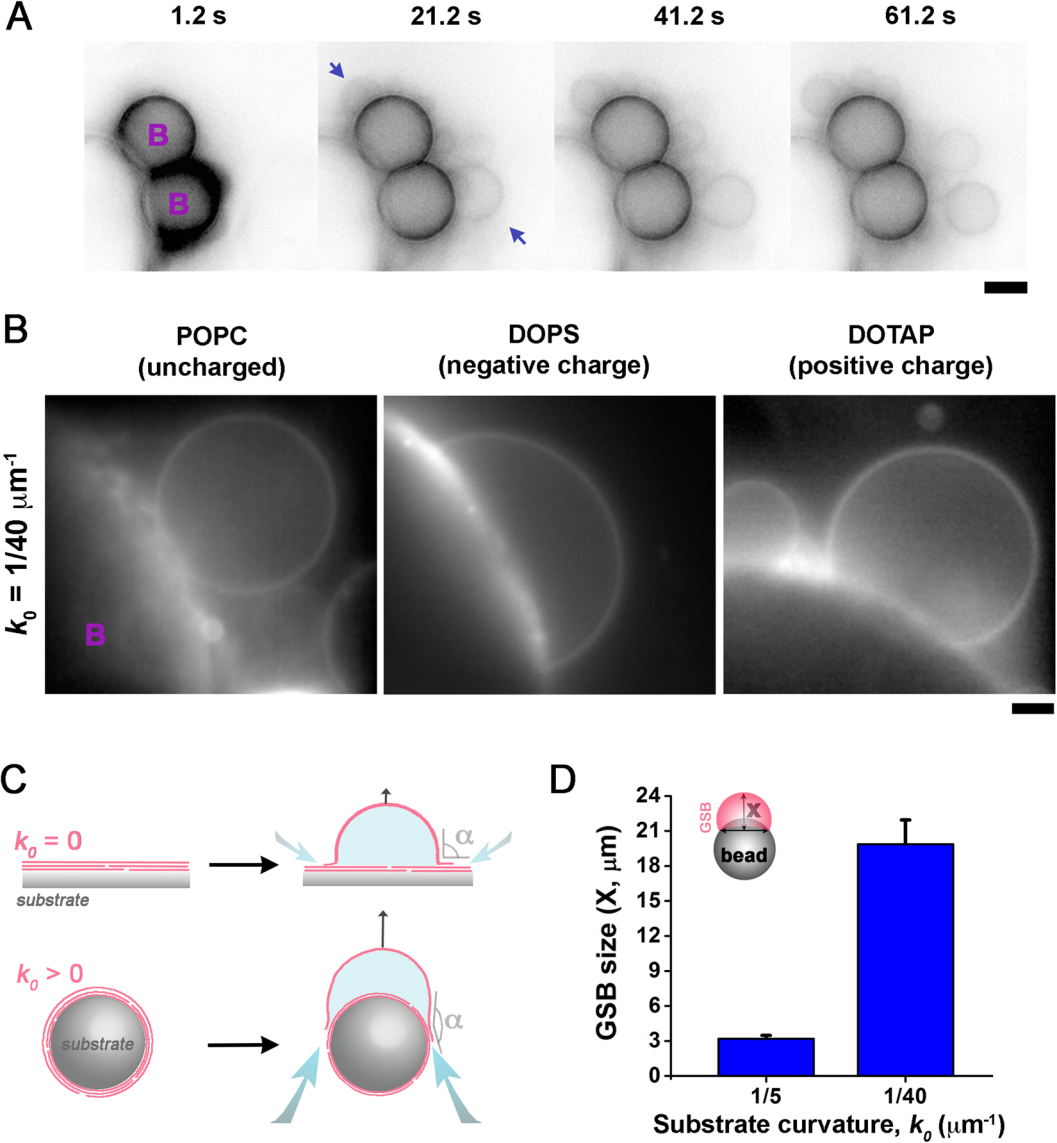
Substrate curvature promotes GSB formation. (**A**) Emergence of GSBs (blue arrows) from lipid films (containing DOPC:DOPE:DOPS:Chol:PI(4,5)P_2_:RhPE 45.5:33:10:10:0.5:1 mol%) deposited on 5 μm silica beads (purple ¨B¨s) during the first minute of hydration in 1 M TRH. Scale bar 2 μm. (**B**) GSB formation from highly charged (99% DOPS and DOTAP) and neutral (99%POPC) lipid films deposited on 40 μm silica beads (purple ¨B¨s) upon 5 minutes of hydration in 1 M TRH. Scale bar 3 μm. (**C**) GSB geometry can be parameterized through the contact angle α between the substrate surface and the GSB membrane. Note that while α ∼ 90° corresponds to a hemisphere on a flat surface (substrate curvature *k*_0_ = 0), it corresponds to an almost complete sphere on a spherical bead (*k*_0_ > 0). (**D**) Dependence of the GSB size, measured as the distance X from the base to the top of GSB (see insert), on the diameter of the silica bead for GSBs made of DOPC:DOPE:DOPS:PI(4,5)P_2_:Chol:RhPE 27:24:15:3:30:1 mol% lipid mixture. Error bars are SD, n = 32 and 36 for 5 and 40 μm beads, respectively.

While gentle hydration of lipid films deposited on several types of surfaces (e.g. borosilicate glass coverslips^41,42^ or Teflon discs^43^) has been reported as a suitable method for GUV production, it is usually restricted to certain lipid compositions and hydration conditions^43,44^. We found that by adding substrate curvature to the hydration equation, the compositional restriction virtually disappears. Lipid films deposited on surfaces with spherical curvatures in the 0.2–0.02 μm^−1^ range (see Methods section, Fig. S1) transform into giant vesicle-like structures (the GSBs) within the first minutes after contact with a disaccharide solution (Figs. 1A and S2). Here we used silica beads with a diameter of 5–40 μm as the substrate of choice for GSB production (Fig. 1). The density and size of the beads allow for easy manipulation as well as proper immobilization of GSBs near the bottom of a microscope observation chamber, enabling the use of high-magnification power objectives (Fig. 1B) for monitoring of GSB membrane remodelling.

We found that GSBs could be produced on silica beads from a variety of complex lipid mixtures (see Table S1, as well as^38,45,46^). Both, highly charged (99 mol% DOPS or DOTAP) and uncharged (99 mol% POPC) lipid films form GSBs on silica beads in 1 M disaccharide (trehalose (TRH) or sucrose) solutions (Fig. 1B). Strikingly, if the films made from these three lipid compositions are deposited on a flat surface, same hydration conditions yield only small multilamellar structures (Fig. S3).

To explain this curvature effect, we parameterize the geometry of the growing GSB by the contact angle (α) between the substrate and the GSB membrane (Fig. 1C). On a flat surface, hydration starts at α = 180° and the angle grows steadily with the GSB emergence. When the GSB becomes a hemisphere, α becomes 90° (Fig. 1C, upper panel), while α < 20° when the GSB growth approaches the final steady state (α is considered 0° for a detached GUV). However, on a curved substrate, α behaves in a different way. As the lamella is initially curved, its swelling and the following GSB growth proceeds without substantial changes in α, leading to the observed correlation between the GSB size and the bead curvature (Fig. 1D). Importantly, α also controls the solute influx into the growing GSB: keeping alpha high facilitates the influx and thus the GSB growth, as will be discussed in details below. We also speculate that the formation of a membrane kink at the GSB-bead boundary interferes with the GSB growth, explaining the correlation between the substrate’s curvature and the swelling process.

### Membrane reservoir: an essential feature of GSBs

**Figure 2:**
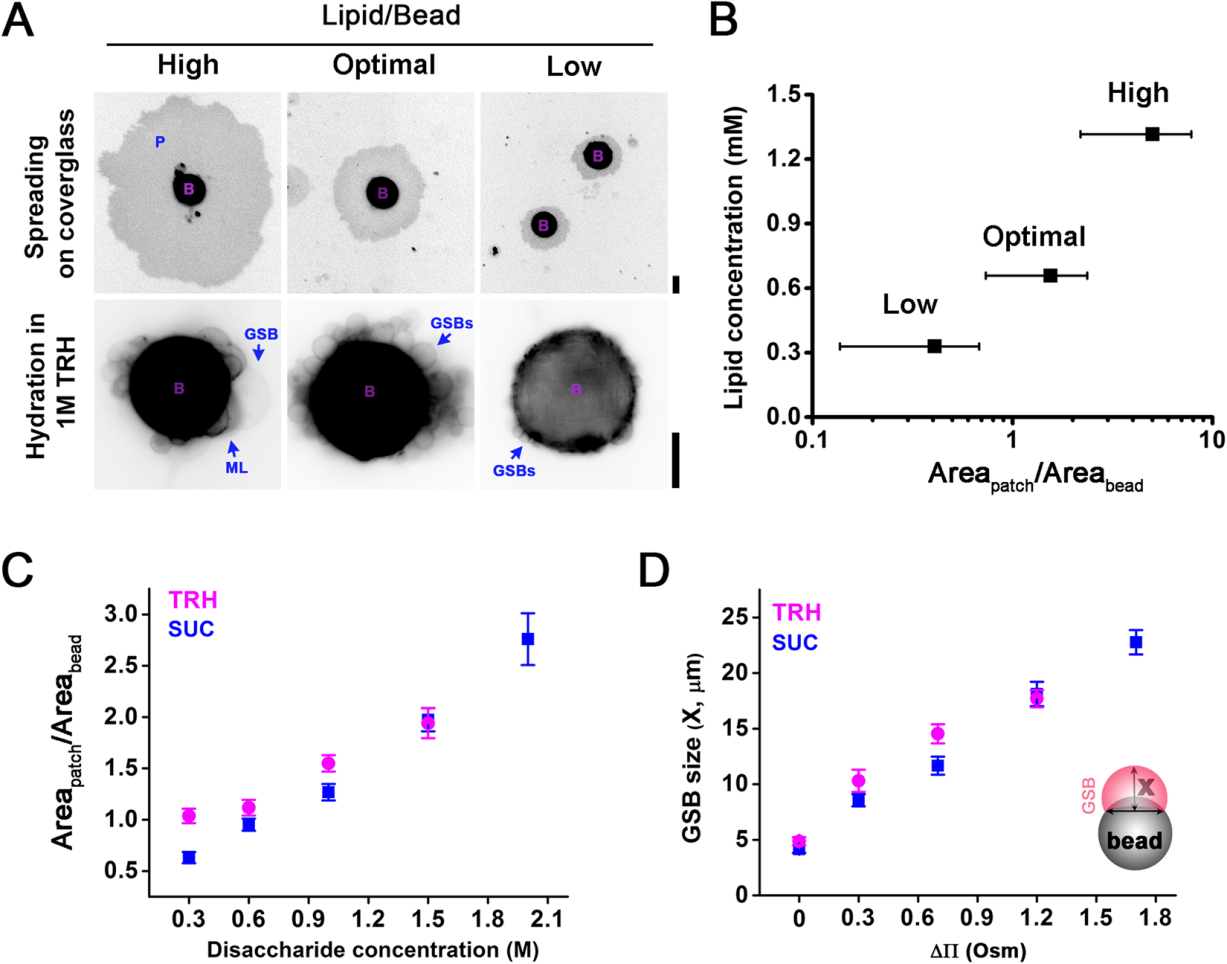
Optimization of GSBs production. (**A**) Membrane patches (P) formed by lipids (99.5:0.5 mol% POPC:RhPE) transferred from 40 μm beads (purple “B”s) to a plasma cleansed coved glass (upper row) and representative images of the corresponding GSB samples formed by hydration in 1 M TRH (lower row). Scale bars are 20 μm. (**B**) Dependence of the lipid to bead ratio (L/B), quantified as the membrane patch area divided by the bead area on the lipid concentration in the initial MLV solution (see Methods). The areas were obtained by analysing images, like those shown in A, with the measuring feature of ImageJ software^55,56^. Error bars show SEM. n > 30 at each point. (**C**) L/B ratio, measured as in B, increases with the concentration of disaccharide solutions used at the prehydration step. Data for POPC:RhPE 99.5:0.5 mol% membrane composition are shown. Error bars are SEM. n > 40 for each point. (**D**) The growth of GSBs induced by osmotic pressure. GSBs prehydrated with different concentrations of disaccharides were subjected to an osmotic shock by their relocation into buffer A, with osmolarity ~300 mOsm. GSB size was estimated as the distance X (see insert) 20 min after GSB transfer to the buffer A. Error bars show SEM, n > 30 for each point.

Apart from the boundary angle, enough membrane reservoir should be available on the substrate to substantiate the GSB growth. A way to measure the size of the membrane reservoir on silica beads was introduced earlier for SUPER templates^27^. Briefly, when a bead touches a clean glass surface, the electrostatic attraction drags the excess lipids from the bead to the glass resulting in the formation of circular membrane patches around the bead (Fig. 2A). We estimated the amount of membrane available on the bead by measuring the ratio between the area of such membrane patches and the total area of the bead (further referred as lipid to bead area ratio or L/B) (Fig. 2A, B and Methods section). We found that the L/B ratio critically affects the GSB formation. With big excess of lipid available on the bead (high L/B), the GSBs emerge mostly as multilamellar formations. However, if the amount of lipid is low GSB formation is aborted altogether. This situation closely resembles the SUPER template, where only a single lipid layer is deposited on the bead surface^27^. Finally, at intermediate L/B ratios, unilamellar GSBs are prevalent (Figs 2A, B, S4 and Table S1). Importantly, the lipid composition affects both, the L/B ratio that yields unilamellar GSBs and the speed of lipid spreading on the glass (Fig. S5). Thus, for each lipid composition, the optimal L/B ratio is to be determined experimentally. Pucadyil and Schmid also reported a correlation between the spread of the lipid on the cover-glass and the concentration of the buffer saline^27^. We followed a similar approach by rehydrating the beads with different concentrations of disaccharides (Fig. 2C) before their deposition to the glass surface for L/B measurements. We observed that bigger concentrations of disaccharide result in bigger spreading of the membrane on the glass (Fig. 2C). Thus, at higher concentrations disaccharides penetrate deeper into the lipid multilayers deposited on the beads, effectively increasing the amount of membrane reservoir available for the GSB production.

TRH is a disaccharide widely described in the literature as a natural protectant of proteins and cellular membranes. It has been implicated in the preservation of the integrity of biological membranes under thermal stress, osmotic shock, and desiccation^47,48^. As fast GSB swelling implies mechanical stress exercised on the lipid membrane, we choose 1 M TRH buffered with 1 mM Hepes, pH 7.0 as the preferred pre-hydration buffer. Importantly, a relocation of the GSBs prehydrated with 1 M TRH into working buffer A (150 mM KCl, 20 mM Hepes, 1 mM EDTA) leads to substantial increase in the GSBs sizes (Fig. 2D). This effect may be due to the difference in osmolarity between 1 M disaccharide solution inside the GSB and the outside buffer, leading to water influx into the GSBs. Indeed, the GSB growth can be further tuned by changing the difference in osmolarity of the media inside and outside the GSBs, i.e. by changing the concentration of the disaccharide at the prehydration step (Fig. 2D).

The GSB growth driven by the water influx indicates that GSB remains connected to the membrane reservoir on the bead. To explore the limits of such GSB swelling, we applied additional osmotic pressure (Fig. S6A) or directly microinjected buffer A into the GSB interior (Fig. S6B). Both approaches resulted in a substantial GSB size growth, confirming a GSB membrane reservoir equivalent to few tens of square microns. The presence of such a reservoir represents a main mechanical difference between the GUV and the GSB membrane templates, making the later mechanically robust^23^.

### GSB’s interior is connected with the external media

**Figure 3:**
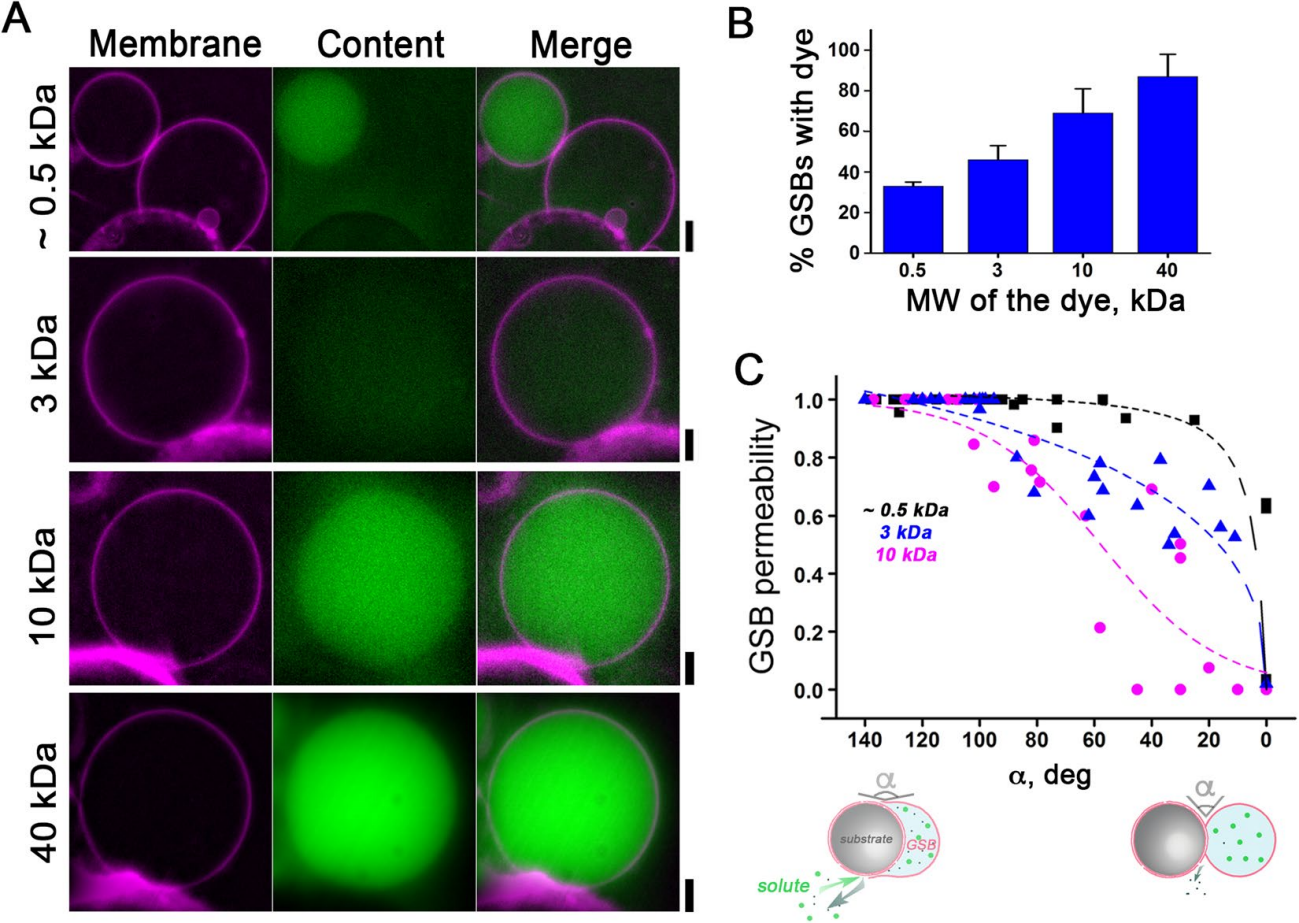
Small but not high MW solutes escape the GSBs. (**A**) GSBs (DOPC:DOPE:DOPS:PI(4,5)P_2_:Chol:RhPE 27:24:15:3:30:1 mol%) produced in the presence of aqueous fluorescent markers of different molecular weights (HiLyte 488 amine TFA salt (~0.5 kDa) < 3 kDa FITC-dextran < 10 kDa FITC-dextran < 40 kDa FITC-dextran). Images were taken 20 minutes after transfer of GSBs (prehydrated in 1 M TRH solution containing the dye) into buffer A. A closed giant vesicle adjacent to a GSB is shown in the upper panel to underline the difference in HiLyte encapsulation between GSB and GUV. Note that 40 kDa FITC-dextran encapsulation was possible only after a two folds increase of the membrane reservoir on the bead. Scale bar 4 μm. (**B**) Percentage of GSBs encapsulating a fluid marker 20 minutes after the GSB transfer into buffer A. Error bars are SEM. n > 50 for the 0.5, 3 and 10 kDa dyes, n = 10 for 40 kDa FITC-Dextran dye. (**C**) The permeability of the GSB contact zone depends on the contact angle α. Permeability was measured as the inverse of fluorescence signal of the dye remaining in the GSBs 20 min after transfer to buffer A normalized to the maximum fluorescence found in GUVs. Dashed lines represent a sigmoidal fit for each data set.

Fast inflation of GSBs by hydrostatic pressure indicates the existence of substantial water permeability through the GSB membrane and/or GSB contact with the lamellas on the bead. To explore whether the water influx is indeed mediated by pores or holes, we prehydrated GSBs in the presence of aqueous fluorescent markers of different hydration radii and then transferred the GSBs into buffer A. HiLyte 488 amine TFA salt (mw 530.45 Da) was absent from the GSB interior after the first 5 minutes of GSB’s transfer into buffer A (Fig. 3A). Slower leakage was observed with 3 kDa FITC-dextran: it disappeared from the GSBs interior 20 min after the transfer (Fig. 3). Bigger markers, such as 10 and 40 kDa FITC-dextrans (with a Stokes radius ~23 Å and 45 Å, respectively) still remained inside the GSBs 1 hour after the transfer to buffer A (Fig. 3A). Interestingly, the presence of 40 kDa-dextrans at the GSB prehydration step significantly decreased the yield of GSB production, indicating impaired penetration of the solutes into interlamellar spaces. Such an inhibitory effect, however, could be partially reversed by augmenting the lipid reservoir on the bead.

These data confirm the existence of a permeability pathway, presumably at the contact zone between the GSB membrane reservoir and the complex lamellar structures on the bead. The encapsulation of big solutes at the initial hydration step of GSB formation and the permanence of such solutes inside the fully grown GSBs indicate that the permeability evolves and decreases during the formation of GSBs. Indeed, GSB permeability is directly related to the contact angle, α (Fig. 3C). When α exceeds 100°, the permeability for big solutes becomes high. When α < 100°, the GSBs interior become virtually non-permeable for molecules with hydration radii bigger than ~20 Å, while smaller solutes can still get in and out of the GSB lumen (Fig. 3C). Notably, TRH used at the prehydration step should escape freely from GSBs, as its molecular weight is below that of HiLyte 488 amine TFA salt and its Stokes radius is below that of 3 kDa FITC-dextran. Hence, upon the GSBs are transferred into a physiologically relevant ionic media, only a minimal amount of disaccharide or other small molecular weight osmoticants used in GSB preparation shall remain in the GSB lumen (<1 mM of TRH, see Fig. S7). Remarkably, sequential immersion of GSBs into different buffers followed by GSBs detachment from the beads enables the creation of GUVs with asymmetric distribution of water-soluble as well as membrane-interacting components. Corroborating this notion, all of the fluorescent markers tested were successfully encapsulated into GUVs formed from GSBs as described (see Methods section, Fig. S8). Hence, this novel method of GUV production allows for easy and fast encapsulation of different solutes into GUVs.

Besides exchange of solutes, the existence of pores or holes at the edge of the GSB reservoir, as well as the lack of direct contact between the GSB membrane and the substrate surface, should mediate the lipid exchange between the two leaflets of the GSB membrane, resulting in symmetrical distribution of lipid species between the leaflets. In accordance with this conjecture, the initial fluorescence of a lipid marker integrated to the membrane of GUVs made from GSBs (see Methods section), decreased to its half upon external addition of sodium dithionite^49^ (Fig. S9).

### Reconstitution of protein-driven membrane remodelling with GSBs

**Figure 4:**
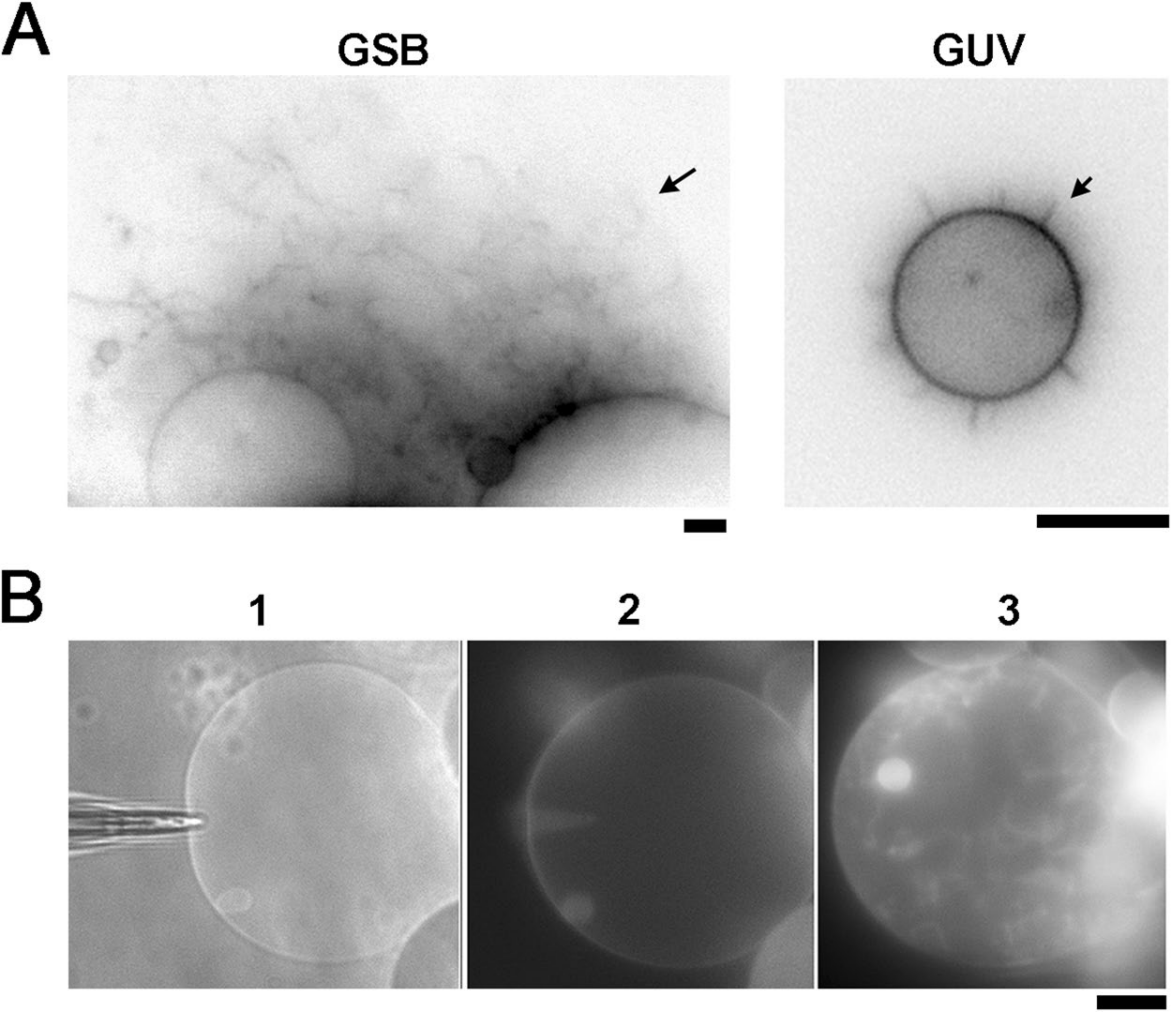
Tubulation of GSB membrane by Dyn1. (**A**) Representative images showing tubulation (indicated by arrows) induced by Dyn1 (0.5 µM) on GSB (left) versus GUV (right). The lipid composition in both cases was DOPC:DOPE:DOPS:PI(4,5)P_2_:Chol:RhPE 58:10:15:2:30:1 mol%. RhPE fluorescence is shown. Scale bar 4 μm. (**B**) Inwards tubulation of a GSB membrane upon microinjection of Dyn1 (0.5 μM) into the GSB lumen. Overlaid bright field/fluorescence image (1) shows the experimental configuration during piercing of the GSB membrane by the injection pipette. Development of the inward membrane tubulation is shown in images (2) and (3). The lipid composition used was DOPC:DOPE:DOPS:PI(4,5)P_2_:Chol:RhPE 27:24:15:3:30:1 mol%. Scale bar 15 μm.

We further tested the GSB system in the reconstitution of membrane remodelling events. The remodelling necessary involves membrane bending. The capability of a protein to bend a membrane and induce membrane curvature has been assessed in the GUV and SUPER template systems by the so-called tubulation assay (e.g., see^29,46^). The essence of tubulation is the transformation of a small piece of a “flat” parent membrane into a highly curved tube upon protein binding. In bulk assays, the whole GUV (or SUPER template) is exposed to the protein, and thus, the tubulation progression depends on the available membrane reservoir. We compared the effect of prototypic curvature-creating protein, Dynamin 1 (Dyn1) on the morphology of GSBs and GUVs made with the same lipid composition. Dyn1 is a well-characterized membrane remodelling protein, capable of creating membrane tubules *in vitro* upon self-assembly on PI(4,5)P_2_ containing membranes^27,46^. As expected, upon Dyn1 addition to the bulk, membrane tubulation activity was robustly detected in GSBs, closely resembling the activity reported with SUPER templates (see Fig. 4A)^46^. GUVs, on the other hand, displayed much reduced tubulation (Fig. 4A), emphasizing the importance of extra membrane reservoir present in GSB and SUPER templates.

Unlike the SUPER, the GSB system also allows analysing tubulation from inside, when Dyn1 is microinjected into the GSB lumen. The microinjection method was previously utilized in GUVs hydrated on flat surfaces^25,50^. Thin glass pipette pierced the GUV membrane and then delivered its contents to the GUV via diffusion/electrophoresis^50,51^. We performed a similar experiment using a thin patch-pipette filled with 0.5 μM Dyn1 solution in buffer A. Shortly upon establishing the connection between the pipette and the GSB interior, inwards membrane tubulation emerged (Fig. 4B), indicating that Dyn1 diffused into the GSB lumen. Dyn1 concentration in the GSB should reach that of the patch-pipette, as such a large protein (100 kDa) is not expected to leak out through the GSB-reservoir contact. These experiments demonstrate that both monolayers of the GSB membrane are accessible for reconstitution of protein-driven membrane remodelling. Thus, unlike GUVs and SUPER templates, GSB enables studying dynamic interactions between molecular players acting from opposite sides of the target membrane in such complex topological membrane transformation as viral infection and multivesicular body formation.

### Whole-GSB patch-clamp configuration allows analysis of nanoscale membrane deformations with GSBs

We next detected that GSBs not only sustain microinjection with a patch-clamp pipette but also enable the use of classical patch-clamping, specifically, establishing the whole-GSB configuration^40^. For that, the glass pipette first touches the GSB membrane to establish a tight contact between its tip and the lipid bilayer. Then, the membrane patch inside the pipette tip is ruptured, and an electrical connection is established between the GSB lumen and the pipette interior^40^. This procedure is essentially equivalent to the “whole-cell” patch-clamping^37^. We routinely used an electrical pulse (Axopatch 200B, ZAP pulse: +1.3 V, 0.5 ms) to rupture the membrane patch, which further confirms the unilamellarity of the GSBs. In the whole-GSB configuration, the GSB interior remains electrically connected to the external media, with the access resistance (R_contact_) ranging between 1 and 50 MΩ (n = 5). Such low electrical resistance is consistent with a diffusional exchange of small solutes between the GSB and external media (see Fig. 3). The whole-GSB configuration was remarkably robust, indicating that GSB stabilization by the membrane reservoir on the bead would enable a mechanical transformation of GSB by the pipette.

**Figure 5:**
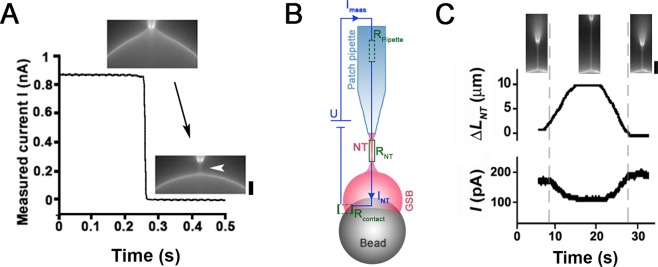
Monitoring of a lipid nanotube (NT) by simultaneous patch-clamp conductance measurements and fluorescence microscopy. (**A**) A representative example of the simultaneous monitoring of the ionic current through the NT lumen and fluorescence microscopy imaging of the NT membrane. GSB membrane contains DOPC:DOPE:DOPS:Chol:PI(4,5)P_2_:RhPE 28.5:25:15:30:1:0.5 mol%. The current drops abruptly at the moment of the NT formation, shown with the arrowhead in the image sequence. Scale bar 3 μm. Holding potential = 10 mV. (**B**) The equivalent circuit of the GSB-NT system. As the resistance of the NT (R_NT_) is much bigger than that of the leakage through the contact between the GSB membrane and the lipid reservoir (R_contact_) as well as that of the pipette (R_pipette_), then I_meas_ ~I_NT_. (**C**) I_meas_ depends inversely on Δ*L*_*NT*_ measured simultaneously with a piezo manipulator and by fluorescence microscopy (the corresponding images are shown above the graphs. Scale bar 4 μm). The dependence of the NT length (*L*_*NT*_) on I_meas_ was further used to calculate the NT radius^52^. Holding potential = 30 mV.

Earlier it has been demonstrated that microinjection and patch-clamp pipettes can be used to pull lipid nanotubes from the respective membrane templates^25,35^. We found that the same technique could be applied in the GSB system. Upon establishing the whole-GSB configuration we slowly pulled the patch-pipette away from the GSB (Fig. 5A). The process was simultaneously monitored by patch-clamp conductance measurements and fluorescence microscopy imaging of the GSB membrane. As the GSB interior is electrically connected to the external media, an initially large ionic current (in nA range) was detected (Fig. 5A). Fluorescence microscopy showed that at the onset of the outward movement of the pipette, the GSB membrane bent forming a catenoidal connection with the pipette tip (Fig. 5A, upper image). With further movement of the pipette the connection collapsed into a cylindrical nanotube^37^ (Fig. 5A, lower image, arrowhead). The collapse was also seen as an acute reduction of the current signal (Fig. 5A). As the access resistance to the NT from both, GSB and pipette sides is much smaller than that of the NT (mOhms versus GOhms), the measured current directly reports the conductance of the NT lumen (Fig. 5B). Such simultaneous optico-electrical measurements allowed to cross-correlate, for the first time, the measurements of the NT conductance with the observation of the NT shape^38,52^. The NT radii obtained from the values of electrical conductance for the NTs pulled from GSB ranged from 150 to 220 nm, indicating low lateral tension of the GSB membrane^52,53^. We expect that such correlative fluorescence and conductance measurements of protein-driven changes of NT geometry will allow characterizing the fast and localized events of membrane constriction and fission by small protein complexes with unprecedented resolution.

To summarize, our initial proof-of-concept experiments indicate that the GSB template should significantly facilitate and further advance our methodological approaches to *in vitro* reconstitution and quantitative analyses of various membrane processes. GSBs are easy to form, friendly to use and virtually non-selective for lipid species. Proteins can be simultaneously added to the interior and exterior of the GSBs, thus allowing for simultaneous reconstitution of membrane processes of different topology. Isolated GSBs can be straightforwardly positioned in the field of view of a powerful microscope, making them easily accessible for correlative electro-optical measurement. GSBs formed on beads of different diameter can be separated in mass for biochemical assays. Finally, GSBs are a good starting material for GUV preparation, as they make GUV preparation fast and easy, allow for solute encapsulation and for overall control of the inner media in the GUVs, and ensure transbilayer symmetry. Thus, we expect GSBs to be included in a common toolkit suitable for the study of membrane mechanics and reconstitution of membrane trafficking events.

## Methods

### Preparation of multilamellar lipid vesicles (MLVs)

1,2-dioleoyl-*sn*-glycero-3-phosphocholine (DOPC), 1,2-dioleoyl-*sn*-glycero-3-phosphoethanolamine (DOPE), 1,2-dioleoyl-*sn-*glycero-3-phospho-L-serine (DOPS), 1-palmitoyl-2-oleoyl-*sn*-glycero-3-phosphocholine (POPC), L-α-phosphatidylinositol-4,5-bisphosphate (PI(4,5)P_2_), 1-hydroxy-2-oleoyl-sn-glycero-3-phosphocholine (OPC), 1,2-dioleoyl-3-trimethylammonium-propane (DOTAP), Sphingomyelin (Brain, Porcine) (brainSM) and 1,2-dioleoyl-*sn*-glycero-3-phosphoethanolamine-*N*-(lissaminerhodamine B sulfonyl) (RhPE) were from Avanti Polar Lipids (Alabaster, AL, USA). Cholesterol was from Sigma-Aldrich (St. Louis, MO). Appropriate amounts of lipid stock solutions in chloroform were mixed in a glass tube to obtain the desired molar compositions (usually at 0.5 to 2 g/L total lipid concentration). The solvent was removed by 30 min of vacuum drying. MLVs were formed by hydration of the lipid film in 1 mM Hepes, pH 7.0 (200 µL final volume). Additional vortexing or pipette mixing was sometimes needed to remove all the lipid material from the walls of the container. The MLV solution could be stored at 4 °C for several days, depending on the lipid composition.

### Formation, variations, and characterization of the GSB template

10 μl of MLVs solution were deposited in 4–5 (approximately) equal drops on a clean Teflon or Parafilm surface (Fig. S1). A small aliquot of a 10% solution of 40 µm plain silica beads (Corpuscular Inc, USA) was three times washed with ultrapure water. Each one of the MLV drops was then gently touched with a 10 μL pipette tip containing 0.2 µL of the beads in water. Note that the beads quickly accumulate at the tip end when the tip is held vertically. The bead-MLV drops were dried in vacuum for 15–30 minutes until complete water evaporation. Dry beads covered by the lipid film could be stored at room temperature in a desiccator cabin for 24 hours. However, immediate use of the beads is preferable. In our experiments, the beads were used (a) to find the optimal bead/lipid ratio, (b) to form the GSBs or (c) to form GUVs.

- *Bead/lipid (B/L) ratio characterization*: the procedure to measure the excess lipid reservoir on the bead have been described elsewhere^27^. Briefly, a cover glass was thoroughly cleansed by sonication in ethanol followed by 10 sec plasma cleaning (Harrick Plasma, USA). Other alternative glass cleansing protocols (such as the use of piranha solution) can be followed^27^. The plasma activated cover glass was inserted into a microscopy chamber (Quick Change Chamber 25 mm Low Profile, Harvard Apparatus) which was then filled with buffer A (150 mM KCL, 10 mM HEPES, 1 mM EDTA). A small portion of the lipid film covered beads from one of the dried drops was transferred into the buffer A in the microscopy chamber. To pick up the beads we used a patch-pipette with fire-closed tip (Fig. S1). Beads went to the bottom of the chamber by gravity and the spreading (spill) of the fluorescently labelled lipid membrane (containing 0.5 mol% of RhPE) around each bead was recorded in real time with an inverted fluorescence microscope (as seen in Figs 2A and S4). Importantly, for lipid compositions containing charged species the time required to achieve a final steady-state size of the spill was longer than that for neutral lipid compositions^54^ (Fig. S4B). The spill area was measured using the ImageJ software^55,56^ to calculate the (bead surface area)/(lipid spill area) ratio (Fig. 2B).
- *Formation of GSBs*: A 10 µL plastic pipette tip was cut from the bottom to approximately 2/3 of its original size. The cut tip was used to take 6 µL of 1 M TRH solution buffered with 1 mM Hepes, pH 7.0. We noticed that, while sucrose could be used instead of TRH, prehydration with 1 M TRH resulted in bigger and more stable GSBs than with 1 M sucrose. Thus, we routinely used TRH for GSB and GUV formation. The tip was carefully detached from the pipette and held in a vertical position all the time. A small portion of the lipid-covered beads from one of the dried drops was picked up and deposited into the tip from the top (Fig. S1). The beads quickly transferred to the bottom of the tip by gravity. The tip was then carefully introduced into a home-made humidity chamber (see Fig. S1) and incubated there for 10–15 minutes at 40–60 °C. Importantly, the incubation time and temperature are to be fine-tunned for each lipid composition. A microscopy chamber with a cover glass cleansed as in (b) and then blocked with Bovine Serum Albumin (BSA) was filled with buffer A and mounted on the stage of an inverted fluorescence microscope. The plastic tip containing the hydrated beads was then carefully withdrawn from the humidity chamber and the end of the tip was put into brief contact with the interface of the buffer in the observation chamber. This way, the beads from the tip dropped down to the bottom of the observation chamber. The amount of the disaccharide solution entering this way into the observation chamber was negligible (Fig. S7). The formation of the GSBs was complete in 10–20 minutes upon the beads contact with the buffer in the observation chamber.
- *Formation of GUVs*: some GUVs always formed along with the GSB in (b). To produce larger amounts of GUVs and separate them from the beads the GSB protocol was slightly modified as follow. The beads with TRH hydrated lamellas were transferred into a microcentrifuge tube containing ~30 µL of the desired GUV buffer (intended to remain in the GUVs interior). The beads were incubated in the buffer with minimal agitation for 20 minutes. Next, the GUVs were separated from the beads and GSBs by gentle shaking or gentle “pipetting” with a wide tip. The beads and GSBs were next left to precipitate by gravity or subjected to gentle centrifugation. The GUVs in the supernatant were transferred to the microscopy chamber with a BSA treated cover glass as in (b) for further observation. To facilitate GUVs observation, 1% (v/v) glycerol can be added to the GUV buffer, so that GUVs precipitate to the bottom of the observation chamber. However, changes in osmotic pressure had to be compensated (1% glycerol corresponds to ~136 mOsm) to avoid osmotic imbalance between GUV interior and external solution.
- *Encapsulation of liquid dyes inside GSBs and GUVs*: liquid dyes are easily internalized into the GSBs when introduced at the TRH hydration stage of the protocol. We used HiLyte 488 amine TFA salt (AnaSpec) at 0.5 g/L and 3, 10 and 40 kDa FITC-Dextrans (Sigma) at 1 g/L dissolved into 1 M TRH with 1 mM Hepes pH 7.0 buffer as the hydration buffer in (b) procedure above. In the case of GUVs, to avoid dye leaking from the GUVs, the dye should be also added to the incubation buffer (that will remain inside the GUVs) during the GSB growth step. Once the GUVs are detached from the bead, they should be transferred to the working buffer without the dye for observation.

### Fluorescence microscopy

Epifluorescence imaging of RhPE-labelled GSB templates was made with Olympus IX-70 inverted microscope (150 × 1.45-NA or 60 × 1.45-NA TIRF objectives) equipped with Andor iXon+ camera (Andor Technology, Belfast, UK) and ImageJ-μManager software package^57^. X-Cite 120Q excitation light source was used in combination with TRITC (554/609 nm) and FITC (475/540 nm) BrightLine single-band filter sets (Semrock) for RhPE and Alexa (HiLyte) based fluorophores, respectively.

Confocal imaging of GUVs and GSBs was performed with TCS SP5 II confocal microscope with 63x/1.20 W objective (Leica Microsystems GmbH). An Argon ion laser was used to excite the samples at 488 and 543 nm, while fluorescence emission was detected at 495–530 and 555–695 nm respectively with Air-cooled R9624 PMTs (Hamamatsu Photonics).

### Injection of solutes into GSBs

Patch-clamp micropipettes were pulled using P2000 puller (Sutter Instruments) from borosilicate glass (GB150–10, Science products) following the manufacturer protocols. The micropipettes were backfilled^58^ with buffer A (Figs 5 and S6B) or 0.5 µM of Dyn1 in buffer A (Fig. 4B). The level of liquid in the pipette was equal or slightly higher than the capillary level to avoid inwards flow of solution. The pipette was inserted into a micropipette holder of a patch-clamp amplifier head (Axopatch 200B (Molecular Devices, Sunnyvale, CA)) with a measuring electrode, located on a microscope stage and connected to a 3D micromanipulator stage (Newport). Upon selection of a GSB in the field of view, the micropipette was moved to the close proximity of the GSB. Next, the GSB membrane was gently touched by the pipette. A tight contact (giga-seal, refs) formed spontaneously between the membrane and the micropipette tip. When needed, slight negative pressure was applied to the pipette to improve the seal^59^. The quality of the seal was assayed through conductance measurements as described elsewhere^37^. A transient electric pulse was applied to the membrane patch inside the pipette using ZAP function of the patch-clamp amplifier. Although negative pressure inside the pipette could be used to break the patch, it was observed that a ZAP pulse (+1.3 V, 0.5 ms) produced more robust rupture of the patch. Once the patch was broken, the interior of the pipette was connected to the interior of the GSB and the protein or buffer from the pipette was injected into the GSB by diffusion or by applying slight positive pressure into the pipette.

### Electro-optical characterization of lipid nanotubes obtained from GSB

Membrane nanotubes were pulled from the GSB membrane following the membrane patch rupture as described earlier^37^. The changes in NT length were simultaneously measured by fluorescence microscopy observation and by piezo micromanipulator. The equivalent electrical circuit for NTs pulled from GSBs is depictured in Fig. 5B. NT conductance was measured in the voltage-clamp mode (1–10 mV/pA gain), at 10–30 mV holding potentials using Axopatch 200B (Molecular Devices, Sunnyvale, CA) amplifier. The signal was digitized using a PC-44 acquisition board (Signalogic, USA) and processed off-line using Origin software (OriginLab, USA) as described earlier^52^. Values of NT radii were obtained by fitting the dependence of the NT conductance of its length as described^52^.

### GSB and GUV membrane tubulation by Dyn1

Dyn1 was purified from Sf9 insect cells transiently transfected with the DNA encoding the protein. Purification was done by affinity chromatography to glutathione S transferase (GST)-tagged Amphiphysin-II SH3 domain^60^. 0.5 μM Dyn1 in buffer A was added to the observation chamber after GSB formation or GUV settling at the cover glass of the microscopy chamber (Harvard Apparatus, USA). Epifluorescence imaging was performed upon 10–15 min of room temperature incubation of the protein with the membrane templates.

## Supplementary information

Supplementary material

## Data Availability

The complete datasets generated during and/or analysed during the study are available from the corresponding author on reasonable request.

## References

[1] Frolov VA, Escalada A, Akimov SA, Shnyrova AV (2015). Geometry of membrane fission. Chem Phys Lipids.

[2] Campelo F, Arnarez C, Marrink SJ, Kozlov MM (2014). Helfrich model of membrane bending: from Gibbs theory of liquid interfaces to membranes as thick anisotropic elastic layers. Adv Colloid Interface Sci.

[3] Mettlen M, Chen PH, Srinivasan S, Danuser G, Schmid SL (2018). Regulation of Clathrin-Mediated Endocytosis. Annu Rev Biochem.

[4] Liu AP, Fletcher DA (2009). Biology under construction: *in vitro* reconstitution of cellular function. Nat Rev Mol Cell Biol.

[5] Walde P, Cosentino K, Engel H, Stano P (2010). Giant vesicles: preparations and applications. Chembiochem.

[6] Sezgin E, Schwille P (2012). Model membrane platforms to study protein-membrane interactions. Mol Membr Biol.

[7] Simunovic M, Bassereau P (2014). Reshaping biological membranes in endocytosis: crossing the configurational space of membrane-protein interactions. Biological chemistry.

[8] Saleem M (2015). A balance between membrane elasticity and polymerization energy sets the shape of spherical clathrin coats. Nature communications.

[9] Shnyrova AV (2007). Vesicle formation by self-assembly of membrane-bound matrix proteins into a fluidlike budding domain. J Cell Biol.

[10] Schmid EM, Richmond DL, Fletcher DA (2015). Reconstitution of proteins on electroformed giant unilamellar vesicles. Methods Cell Biol.

[11] Shi Z, Baumgart T (2015). Membrane tension and peripheral protein density mediate membrane shape transitions. Nat Commun.

[12] Aimon S (2014). Membrane shape modulates transmembrane protein distribution. Developmental cell.

[13] Witkowska A, Jahn R (2017). Rapid SNARE-Mediated Fusion of Liposomes and Chromaffin Granules with Giant Unilamellar Vesicles. Biophysical journal.

[14] Dimova R (2006). A practical guide to giant vesicles. Probing the membrane nanoregime via optical microscopy. *Journal of physics*. Condensed matter: an Institute of Physics journal.

[15] Döbereiner H-G, Evans E, Kraus M, Seifert U, Wortis M (1997). Mapping vesicle shapes into the phase diagram: A comparison of experiment and theory. Phys. Rev. E.

[16] de Almeida RF, Loura LM, Prieto M (2009). Membrane lipid domains and rafts: current applications of fluorescence lifetime spectroscopy and imaging. Chemistry and physics of lipids.

[17] Baumgart T (2007). Large-scale fluid/fluid phase separation of proteins and lipids in giant plasma membrane vesicles. Proceedings of the National Academy of Sciences of the United States of America.

[18] Evans E, Rawicz W (1990). Entropy-driven tension and bending elasticity in condensed-fluid membranes. Phys Rev Lett.

[19] Korlach J, Reichle C, Muller T, Schnelle T, Webb WW (2005). Trapping, deformation, and rotation of giant unilamellar vesicles in octode dielectrophoretic field cages. Biophys J.

[20] Yamada A, Lee S, Bassereau P, Baroud CN (2014). Trapping and release of giant unilamellar vesicles in microfluidic wells. Soft Matter.

[21] Svoboda K, Block SM (1994). Biological applications of optical forces. Annu Rev Biophys Biomol Struct.

[22] Streicher P (2009). Integrin reconstituted in GUVs: a biomimetic system to study initial steps of cell spreading. Biochim Biophys Acta.

[23] Bhatia T, Agudo-Canalejo J, Dimova R, Lipowsky R (2018). Membrane Nanotubes Increase the Robustness of Giant Vesicles. ACS Nano.

[24] Figard L, Sokac AM (2014). A membrane reservoir at the cell surface: unfolding the plasma membrane to fuel cell shape change. Bioarchitecture.

[25] Karlsson M (2002). Formation of geometrically complex lipid nanotube-vesicle networks of higher-order topologies. Proc Natl Acad Sci USA.

[26] Billerit C (2011). Heat-induced formation of single giant unilamellar vesicles. Soft Matter.

[27] Pucadyil TJ, Schmid SL (2010). Supported bilayers with excess membrane reservoir: a template for reconstituting membrane budding and fission. Biophys J.

[28] Neumann S, Pucadyil TJ, Schmid SL (2013). Analyzing membrane remodeling and fission using supported bilayers with excess membrane reservoir. Nat Protoc.

[29] Pucadyil TJ, Schmid SL (2008). Real-time visualization of dynamin-catalyzed membrane fission and vesicle release. Cell.

[30] Roux A (2013). The physics of membrane tubes: soft templates for studying cellular membranes. Soft Matter.

[31] Waugh RE (1982). Temperature dependence of the yield shear resultant and the plastic viscosity coefficient of erythrocyte membrane. Implications about molecular events during membrane failure. Biophysical journal.

[32] Evans E, Yeung A (1994). Hidden dynamics in rapid changes of bilayer shape. Chem Phys Lipids.

[33] Heinrich M, Tian A, Esposito C, Baumgart T (2010). Dynamic sorting of lipids and proteins in membrane tubes with a moving phase boundary. Proc Natl Acad Sci USA.

[34] Prevost, C., Tsai, F. C., Bassereau, P. & Simunovic, M. Pulling Membrane Nanotubes from Giant Unilamellar Vesicles. *J Vis Exp*, 10.3791/56086 (2017). 10.3791/56086 PMC5755540 29286431

[35] Evans E, Bowman H, Leung A, Needham D, Tirrell D (1996). Biomembrane templates for nanoscale conduits and networks. Science.

[36] Jesorka A (2011). Generation of phospholipid vesicle-nanotube networks and transport of molecules therein. Nat Protoc.

[37] Frolov VA, Lizunov VA, Dunina-Barkovskaya AY, Samsonov AV, Zimmerberg J (2003). Shape bistability of a membrane neck: a toggle switch to control vesicle content release. Proc Natl Acad Sci USA.

[38] Shnyrova AV (2013). Geometric catalysis of membrane fission driven by flexible dynamin rings. Science.

[39] Garten, M., Aimon, S., Bassereau, P. & Toombes, G. E. Reconstitution of a transmembrane protein, the voltage-gated ion channel, KvAP, into giant unilamellar vesicles for microscopy and patch clamp studies. *J Vis Exp*, 52281, 10.3791/52281 (2015). 10.3791/52281 PMC4354550 25650630

[40] Garten M (2017). Whole-GUV patch-clamping. Proc Natl Acad Sci USA.

[41] Wegrzyn I (2011). Membrane protrusion coarsening and nanotubulation within giant unilamellar vesicles. J Am Chem Soc.

[42] Reeves JP, Dowben RM (1969). Formation and properties of thin-walled phospholipid vesicles. J Cell Physiol.

[43] Rodriguez N, Pincet F, Cribier S (2005). Giant vesicles formed by gentle hydration and electroformation: a comparison by fluorescence microscopy. Colloids Surf B Biointerfaces.

[44] Akashi K, Miyata H, Itoh H, Kinosita K (1996). Preparation of giant liposomes in physiological conditions and their characterization under an optical microscope. Biophys J.

[45] Carravilla P, Nieva JL, Goni FM, Requejo-Isidro J, Huarte N (2015). Two-photon Laurdan studies of the ternary lipid mixture DOPC:SM:cholesterol reveal a single liquid phase at sphingomyelin:cholesterol ratios lower than 1. Langmuir.

[46] Mattila JP (2015). A hemi-fission intermediate links two mechanistically distinct stages of membrane fission. Nature.

[47] Newman YM, Ring SG, Colaco C (1993). The role of trehalose and other carbohydrates in biopreservation. Biotechnol Genet Eng Rev.

[48] Harrigan PR, Madden TD, Cullis PR (1990). Protection of liposomes during dehydration or freezing. Chem Phys Lipids.

[49] Steinkuhler J, De Tillieux P, Knorr RL, Lipowsky R, Dimova R (2018). Charged giant unilamellar vesicles prepared by electroformation exhibit nanotubes and transbilayer lipid asymmetry. Sci Rep.

[50] Wick R, Angelova MI, Walde P, Luisi PL (1996). Microinjection into giant vesicles and light microscopy investigation of enzyme-mediated vesicle transformations. Chem Biol.

[51] Ferrer-Tasies L (2013). Quatsomes: vesicles formed by self-assembly of sterols and quaternary ammonium surfactants. Langmuir.

[52] Bashkirov PV (2008). GTPase cycle of dynamin is coupled to membrane squeeze and release, leading to spontaneous fission. Cell.

[53] Kummrow M, Helfrich W (1991). Deformation of giant lipid vesicles by electric fields. Phys Rev A.

[54] Ainla A, Gozen I, Hakonen B, Jesorka A (2013). Lab on a Biomembrane: rapid prototyping and manipulation of 2D fluidic lipid bilayers circuits. Sci Rep.

[55] Schneider CA, Rasband WS, Eliceiri KW (2012). NIH Image to ImageJ: 25 years of image analysis. Nat Methods.

[56] Schindelin J (2012). Fiji: an open-source platform for biological-image analysis. Nat Methods.

[57] Edelstein, A., Amodaj, N., Hoover, K., Vale, R. & Stuurman, N. Computer control of microscopes using microManager. *Curr Protoc Mol Biol* **Chapter 14**, Unit14 20, 10.1002/0471142727.mb1420s92 (2010). 10.1002/0471142727.mb1420s92 PMC3065365 20890901

[58] Komarova Y., Peloquin J., Borisy G. (2007). Preparation and Loading of Protein Samples for Microinjection. Cold Spring Harbor Protocols.

[59] Tunuguntla RH, Escalada A, A Frolov V, Noy A (2016). Synthesis, lipid membrane incorporation, and ion permeability testing of carbon nanotube porins. Nature Protocols.

[60] Stowell MHB, Marks B, Wigge P, McMahon HT (1999). Nucleotide-dependent conformational changes in dynamin: evidence for a mechanochemical molecular spring. Nature Cell Biology.

