# Supplementary material for: Combining patch-clamping and fluorescence microscopy for quantitative reconstitution of cellular membrane processes with Giant Suspended Bilayers
Source: Sci Rep. 2019 May 10;9:7255. doi: 10.1038/s41598-019-43561-4 (PMC6510758; doi:10.1038/s41598-019-43561-4)
Supplement: Supplementary file 1 — Supplementary material [file 41598_2019_43561_MOESM1_ESM.pdf]

Combining patch-clamping and fluorescence microscopy for quantitative reconstitution of cellular membrane processes with Giant Suspended Bilayers

Ariana Velasco-Olmo[1], Julene Ormaetxea Gisasola[1], Juan Manuel Martinez Galvez[1], Javier Vera Lillo[1], Anna V. Shnyrova[1]*

[1]Biofisika Institute (CSIC, UPV/EHU) and Department of Biochemistry and Molecular Biology, University of the Basque Country, Bilbao, Spain

**Correspondence to anna.shnyrova@ehu.eus*

This Supplementary file contains:

Supplementary Figures S1 – S9
Supplementary Table S1
Supplementary References

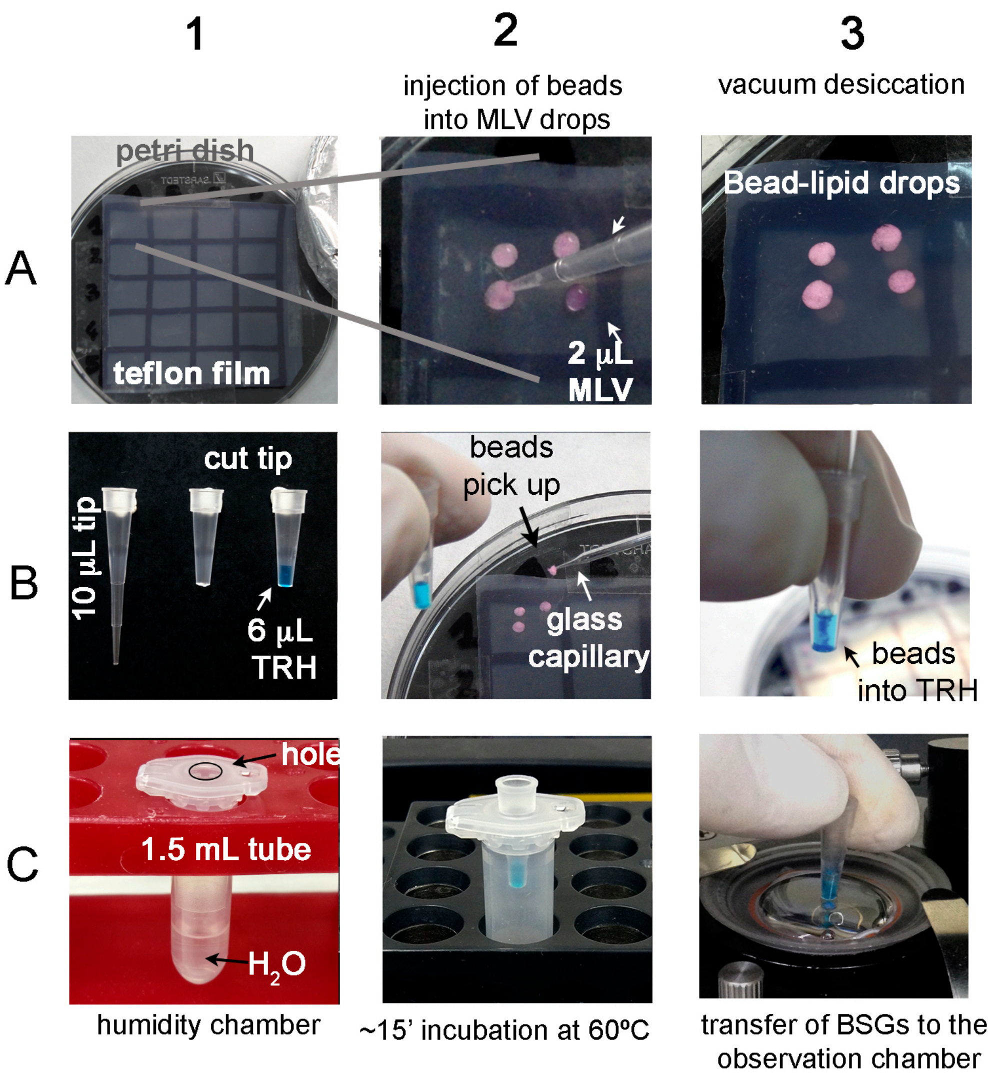

Figure S1. Steps of GSB formation. **A1**. A clean Teflon or Parafilm surface film is firmly attached to the bottom of a plastic petri dish. **A2.** 10 µl of MLVs solution is placed in ~ 4-5 equal drops on the Teflon (in the case shown here, the MLVs contain a RhPE dye, giving them a pink appearance). A small aliquot of a 10% solution of 40 µm silica beads (Corpuscular Inc., USA), three times washed with ultrapure water, is used for the lipid deposition. Each one of the MLV drops is touched with a pipette tip containing 0.2 µL of the beads in water (giving a white tone to the MLV drops, as depicted). **A3.** The drops containing the bead-MLV mixture are dried in vacuum for until water is completely evaporated. The dry beads are further used to analyse bead/lipid ratio, form GSBs or GUVs. **B1.** A 10 µL plastic pipette cut at approximately 2/3 of its length is used to take 6 µL of 1M TRH solution buffered with 1 mM Hepes. If encapsulation of a solute or aqueous fluorescence marker into GSBs is required, it is added to the TRH solution at this step. In the case depicted, 1M TRH was doped with 0.5 g/L of HiLyte 488 amine TFA salt, which gave the blue colour to the solution. **B2**. A small portion of the lipid-covered beads from one of the dried drops in (C3) is picked up with a fire-closed patch glass capillary. **B3**. The beads are deposited into the cut tip containing the TRH solution from the top of the tip. **C1**. An in-house humidity chamber is shown, made from a 1.5 mL microcentrifuge tube (or a 50mL centrifuge tube) by making a hole in its tap and filling it halfway with distilled water. Importantly, the tap hole diameter should allow for a tight fit of the cut tip, as

shown in **C2.** The tip is carefully introduced into the home-made humidity chamber which then is transferred to a hot plate and left for 10-20 minutes at 40-60°C. **C3**. A microscopy observation chamber with a clean, BSA blocked cover glass is filled with buffer A and mounted on an inverted microscope stage. The plastic tip with the beads is withdrawn from the humidity chamber and put into brief contact with the surface of the buffer in the observation chamber. At this point, the level of liquid in the tip rises by capillarity, while the beads drop down to the bottom of the observation chamber by gravity. The amount of the disaccharide solution entering the observation chamber is negligible (see Figure S7). Formation of the GSBs begins immediately after the bead transfer into the observation chamber. GSBs growth is complete in less than 20 minutes upon the first contact of the beads with the buffer solution.

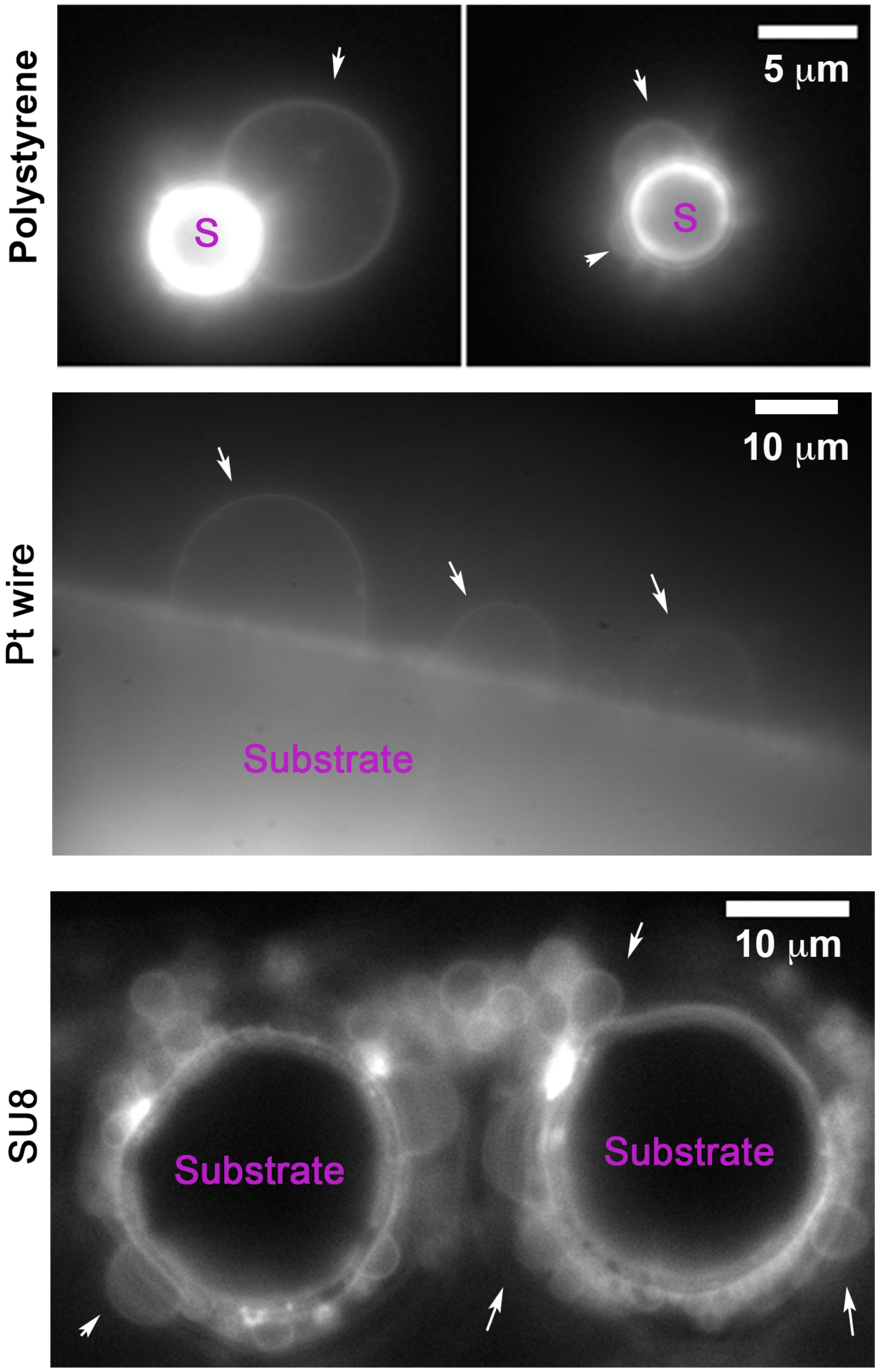

Figure S2. **GSBs formation takes place on various substrates with curvatures in the 0.2-0.02 μm^{-1} range**. GSBs form on 5 μm polystyrene beads (upper row), 50 μm platinum wire (center row), and 20 μm SU8 pillars vertically grown on a cover slip.

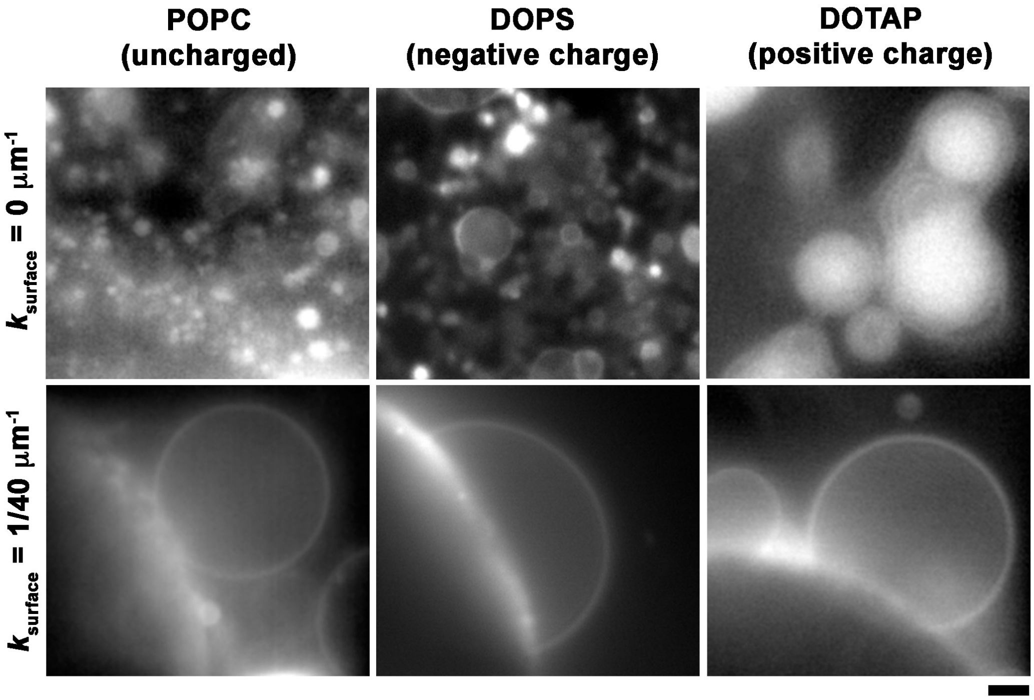

Figure S3. Comparison of the GSB formation on flat ($k_{surface}$ = 0) and curved surfaces ($k_{surface}$ > 0). Images correspond to spontaneous hydration of different lipid films on a flat cover glass (upper row) and on the 40 µm beads 5 minutes after addition of 1M TRH (lower row). Scale bar 3 µm.

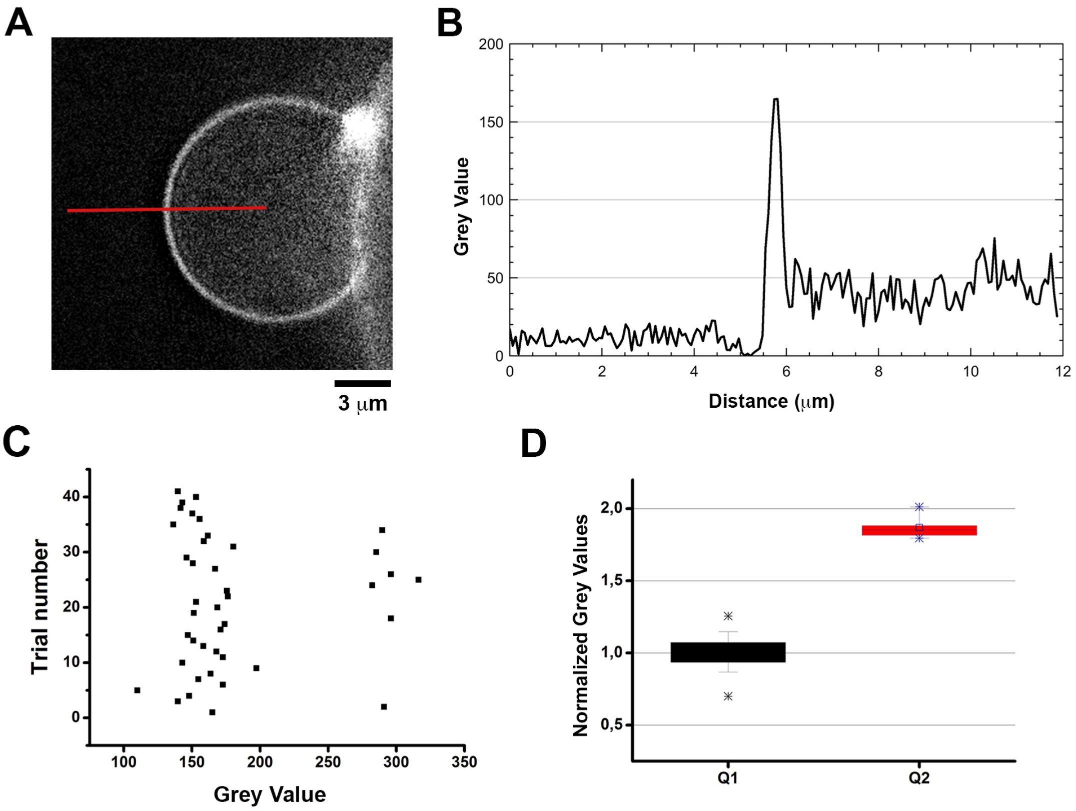

Figure S4. Analysis of GSB lamellarity. A. Confocal image of the equatorial plane of a GSB. Background fluorescence was subtracted using the rolling ball algorithm of ImageJ software[1] **B**. Fluorescence intensity profile along a line (red line in A) crossing the GSB membrane. **C**. The distribution of the peak fluorescence intensities obtained from the profiles shown in B. Each point of the graph corresponds to the mean peak intensity measured with three different plot profiles drawn randomly across a single BSG. Two populations with lower and higher intensities are clearly seen. **D**. Box plot of the data from C normalized by the mean value of the population with the lower grey value. Q1 corresponds to unilamellar BGSs, while BGSs in Q2 have double lamellas. Error bars show SD.

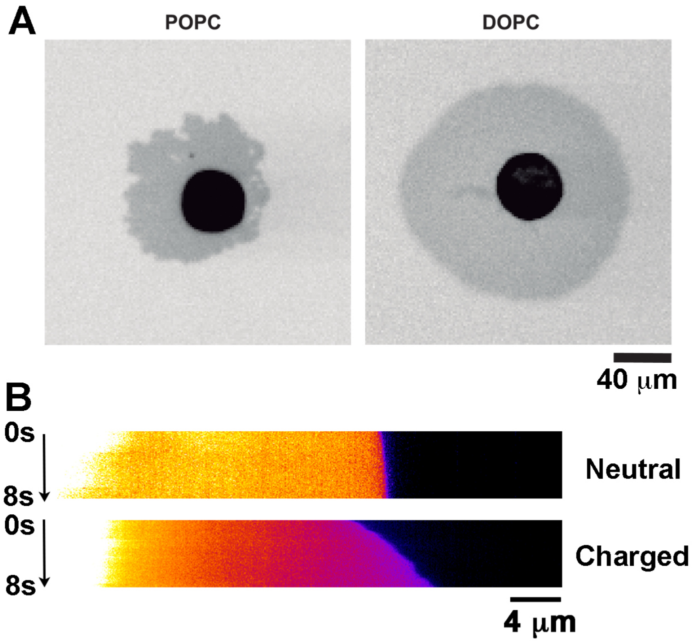

Figure S5. The transfer (spill) of excess lipid reservoir from BGSs to coverglass varies with the lipid composition. **A**. Images of lipid spills as in Figure 2A obtained from the beads covered by the same amount of MLVs made from POPC:RhPE and DOPC:RhPE at 99:1 mol%, respectively. The contrast was inverted for clarity. **B.** Kymographs showing the different kinetics of lipid spilling from the bead to the cover glass for neutral (99:1mol% DOPC:RhPE) versus highly charged lipid composition (68:20:10:1:1mol% DOPC:DOPS:Chol:PI(4,5)P_2:RhPE)[2].

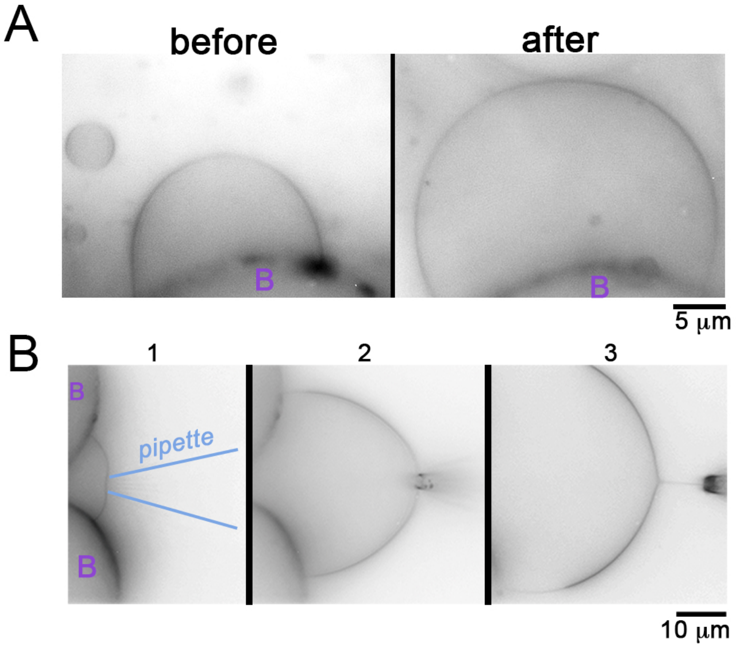

Figure S6. BGSs inflation by hydrostatic pressure reveals extensive membrane reservoir. **A.** GSB (DOPC:DOPS:Chol:Rh-DOPE 69:20:10:1 mol%) formed in buffer A before and after perfusion with 1 mM Hepes, pH 7.0. The hypotonic shock induces an influx of water into the GSB, which augments its radius ~2 times translated into a four-fold increase of its area. **B.** Injection of buffer from a patch-pipette into a GSB (made of DOPC:DOPE:Chol:RhPE 60:29:10:1 mol%) leads to ~16 fold increase of the GSB area. In both panels, contrast has been inverted for clarity. Purple "B"s indicates the bead location.

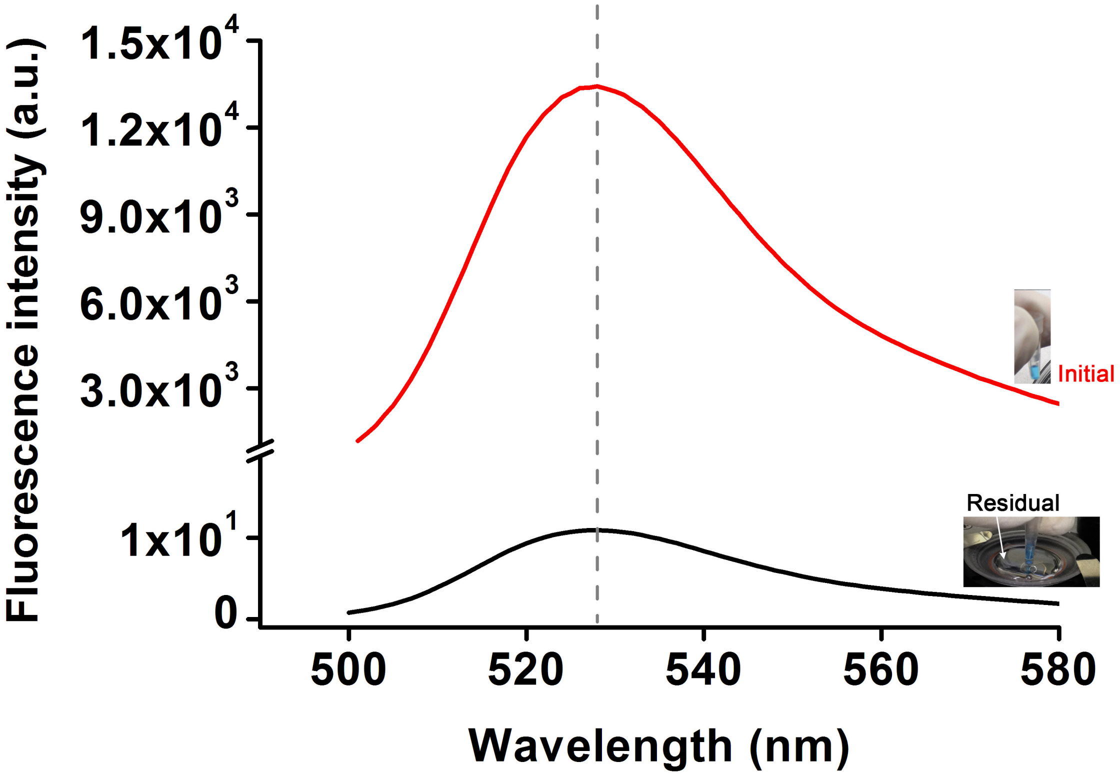

Figure S7. Estimation of TRH amount transferred into the observation chamber with GSBs. The initial emission spectrum of a mixture of 1M TRH mixed with 1g/L of 10 kDa FITC-Dextran was measured in a spectrophotometer (excitation 490 nm). A pipette tip (cut as in the GSB protocol) was filled with 6 μL of the mixture. The tip was placed in brief contact with the surface of 1 mL of buffer A, similarly to the corresponding step in the GSB protocol. The residual dextran and TRH transferred to the buffer A were estimated by comparing the initial emission value at 528 nm to that of the resulting mixture in buffer A (black trace). The concentration of the transferred TRH was estimated as 0.81±0.3 mM (n=4, SD is shown). If a lower concentration of TRH is required, the observation chamber is to be perfused with the desired solution several times to eliminate TRH traces.

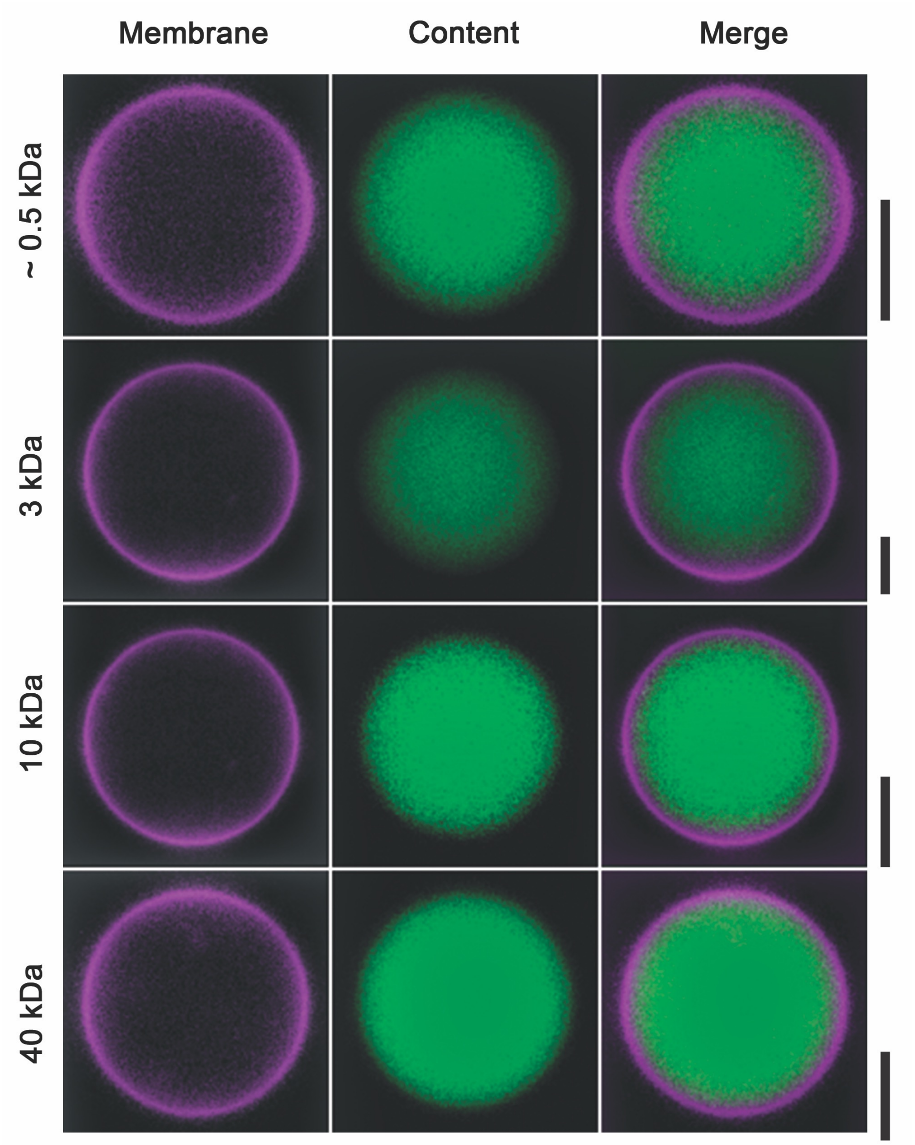

Figure S8. Encapsulation of aqueous dyes (FITC-Dextrans) into GUVs formed from GSBs (see Methods). Shown are the epifluorescence images with the subtracted background. Scale bars are 5 μm.

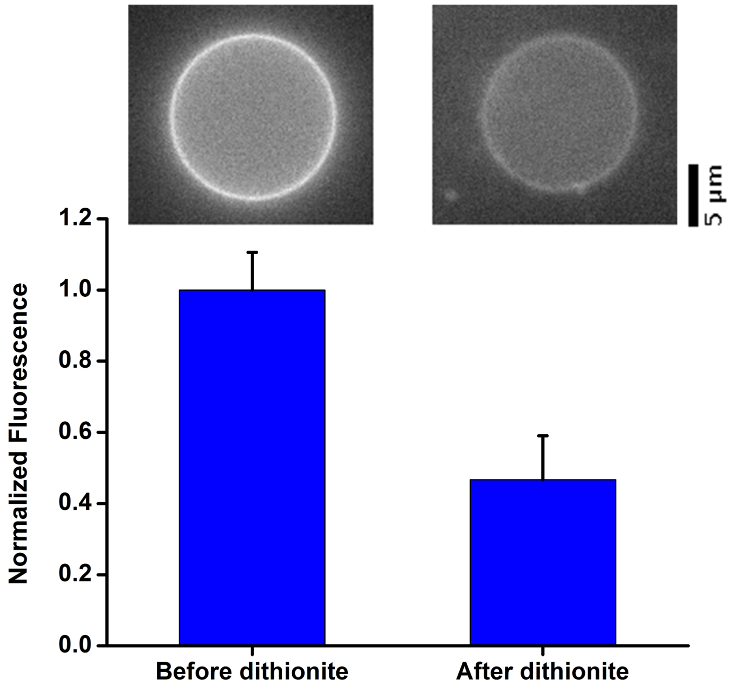

Figure S9. Symmetry of GSBs leaflets. The initial fluorescence of 1mol% RhPE integrated into the GUV membrane decreases to its half during the first 5 minutes upon addition of 5 mM sodium dithionite to the external solution. GUVs were prepared from GSBs following the protocol described in Methods. n=20. Error bars are SD.

Membrane composition	Molar ratio, %	Total lipid, g/L
POPC	100	1
DOPC	100	0.5
DOPC:DOPE	66:34	1
DOPC:DOPE:Chol	40:30:30	1
DOPC:Chol	70:30	1
DOPC:DPPC:Chol	35:35:30	1
DOPC:DOPS	70:30	1
DOPC:DOPS:Chol	45:25:30	1
DOPC:DOPE:DOPS:Chol	30:30:30:10	1
DOPC:DOPE:DOPS:Chol	30:25:15:30	1
DOPC:DOPE:DOPS:Chol:PI(4,5)P_2	27:26:15:30:2	0.5
DOPC:DOPE:DOPS:Chol:PI(4,5)P_2	10:45:15:30:5	1.5
DOPS	100	1
DOPS:Chol	90:10	1
DOPC:Chol:OPC	40:30:30	1
DOPC:Chol:OPC	40:40:20	1
Chol:OPC	50:50	1
DOPC:DOPS:brainSM	50:30:20	1
DOTAP	100	1

Table S1. Total lipid/100 μL of initial MLV stock resulting in optimal GSBs production of the lipid compositions tested. For GSB preparation ~2μL of MLV stock was mixed with ~0.2 μL of bead precipitate.

Supplementary References

1 Schneider, C. A., Rasband, W. S. & Eliceiri, K. W. NIH Image to ImageJ: 25 years of image analysis. *Nat Methods* **9**, 671-675 (2012).

2 Ainla, A., Gozen, I., Hakonen, B. & Jesorka, A. Lab on a Biomembrane: rapid prototyping and manipulation of 2D fluidic lipid bilayers circuits. *Sci Rep* **3**, 2743, doi:10.1038/srep02743 (2013).
